# Use of the electronic nose to monitor the influences of modified atmosphere packaging on the storage of contaminated garlic

**DOI:** 10.1016/j.heliyon.2025.e42609

**Published:** 2025-02-12

**Authors:** Alireza Makarichian, Ebrahim Ahmadi, Reza Amiri Chayjan, Doostmorad Zafari, Seyed Saeid Mohtasebi

**Affiliations:** a Department of Biosystems Engineering, Faculty of Agriculture, Bu-Ali Sina University, Hamadan, Iran; b Department of Plant Protection, Faculty of Agriculture, Bu-Ali Sina University, Hamadan, Iran; c Department of Agricultural Machinery Engineering, College of Agriculture and Natural Resources, University of Tehran, Karaj, Iran

**Keywords:** Electronic nose, MAP, Nano-packaging, Quality, Storage

## Abstract

Garlic is a valuable product that has an excessive application due to its impressive nutritional value. The issue of garlic storage as a seasonal product is critical. Therefore, the effective conditions in the storage should be identified and monitored. In this research, three important treatments in garlic storage including packaging materials, packaging atmosphere, and fungal infection were studied. The effect of these treatments on some important qualitative indices in garlic such as weight reduction, browning index, acidity, total soluble solids, toughness, etc was investigated. Also, the non-destructive technology of the electronic nose (E-Nose) was used as a complementary solution in garlic quality monitoring. The data were evaluated by the Analysis of Variance (ANOVA), Principal Component Analysis (PCA), Backpropagation Neural Network (BPNN), Linear Discriminant Analysis (LDA), and Partial Least-Squares Regression (PLSR) methods. The results disclosed well that applying the studied treatments can affect the changes in qualitative traits. Also, changes in qualitative traits were associated with aromatic changes. The E-Nose responses toward the aroma of different treatment combinations (TCs) had a unique pattern but did not represent an exclusive trend. The PLSR results proved among the qualitative properties, the physical and mechanical traits had the highest (97 %) and lowest (24 %) correlation with aroma changes, respectively. Therefore, the E-Nose can be employed as a complementary and non-destructive solution in the quality monitoring of the storage of products such as garlic.

## Highlights

• Garlic was inoculated to three fungal pathogens.
• The samples were stored in two types of packaging and under MAP conditions.
• A composition of 1 % O_2_, 5 % CO_2_, and 94 % N_2_ was used in MAP.
• The E-Nose was used to assess the aromas' profiles of each treatment composition.
• The changes in qualitative traits were inspected.

## Graphical abstract

**Figure:**
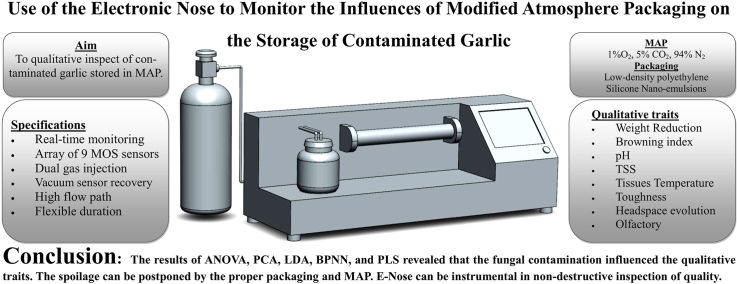

**Practical applications**

Garlic inoculated to three fungal pathogens. The samples were stored in two types of packaging and under MAP conditions. A composition of 1 % O_2_, 5 % CO_2_, and 94 % N_2_ was used in MAP. The E-Nose was used to assess the aromas’ profiles of each treatment composition. The changes in qualitative traits were inspected. The ANOVA, PCA, LDA, BPNN, and PLSR were implemented to analyze the obtained data.

## 1 Introduction

Garlic (*Allium sativum* L.) has been cultivated for its flavorful bulbs and is highly prized for its nutritional and medicinal properties [1]. In recent years, garlic production has faced new sources of postharvest losses that have put its quality at risk such as pathogenic contaminations, pests, nematodes, and physiological disorders. Pathogenic contamination can cause genetic and structural abnormalities. This case is further intensified by post-harvest handling processes such as drying, storage, transportation, and marketing. Although many pathogens and microorganisms initiate their infection through plant growth, the resulting diseases can progress during the postharvest period.

One of the invasive pathogens for garlic is fungal pathogens. Garlic is subject to damage by several fungi such as *Alternaria porri*, *Aspergillus niger*, *Botrytis allii*, *Colletotrichum circinans*, *Fusarium* spp.*, oxysporum f. sp. Cepae*, *Penicillium* spp., and *Sclerotium cepivorum* [2]. The diseases caused by the mentioned fungi can present symptoms such as waxy breakdown. The waxy breakdown is the deterioration of the outer cloves of garlic so that the damaged tissue becomes dark yellow, soft, transparent, and sticky. Many of these fungi produce a wide range of toxins such as *moniliformin*, *fusaproliferin*, *fusaric* acid, *beauvericin*, and *fumonisins*. Most of these mycotoxins remain alive chemically during food processing, which poses a major health risk. Therefore, there is a strong need to identify and store the damaged products in optimal conditions to maintain their integrity [3].

Garlic storage is used to relieve stresses relating to the inconsistency of the food supply in the face of natural and inevitable variability. This technique permits garlic to be consumed for a more extended period. Poor storage conditions can play a key role in inducing stresses, degrading the qualitative properties, and predisposing to shorter shelf-life. The loss associated with postharvest storage is mainly caused by physiological disorders, pathogenic infection, mechanical processing, and the lack of efficient storage technologies [4]. These losses can be lessened by new solutions such as new packaging material utilization, modified atmosphere packaging, and non-destructive separation of contaminated products [5].

The primary purposes of food packaging as well as the MAP are quality consistency, protection, storage period elongation, and minimizing the decay procedure of perishable foods [6,7]. Food may interact differently with the diverse packaging materials and environments, which may have dissimilar effects on preserving product quality [8]. Previous research clarified the great effectiveness of different storage environments on the rate of structural deterioration, sprouting, appearance characteristics, and consequently the quality of a product such as garlic [4].

Garlic contains a large amount of *allicin*, which has a high potential for hypoglycaemic and bactericidal activity. *Allicin* is unstable and a by-product of *allin* decomposition, which quickly converted to fat-soluble compounds such as *diallyl sulfide*, *diallyl disulfide*, *diallyl trisulfide*, *allyl methyl trisulfide*, and *diallyl tetrasulfide* [9]. Therefore, the slightest loss in the quality of garlic and its texture's stability leads to significant fluctuations in its aroma. The aroma variation of garlic during storage can be used as a non-destructive index to correlate the influences of storage factors.

Several studies aimed to study the volatile organic compounds (VOCs) by typical techniques such as high-performance liquid chromatography [10] and gas chromatography-mass spectrometry [11]. These approaches are costly, resource-intensive, and not suited for online usage. Dissimilar to the mentioned approaches, the E-Nose provides a substitute solution to investigating volatile fingerprints like the biological olfaction system [12]. The E-Nose has a broad range of applications in the food and agricultural industry [[13], [14], [15], [16], [17]]. Some advantages of the E-Nose are user-friendly function, high sensitivity, inexpensiveness, and rapidity. The functional principle in typical approaches is established on specific VOCs, while the E-Nose is organized by several non-selective sensors that react against odor molecules. To model and authorize novel supervising systems such as E-Nose, it is compulsory to utilize machine learning approaches and evaluation metrics to be concerned with enormous amounts of diverse data and draw out beneficial knowledge from them. Some tasks of machine learning algorithms and evaluation metrics implicated in research include the creation of practical patterns, recognition of deviations and inconsistencies, or differentiation and categorizing of scenarios. Therefore, E-nose data demonstrate some urges for each of the related phases concerned in the design of a machine learning system.

Taking the above into consideration, this research aimed to (1) consider the effect of the packaging atmosphere, the packaging material, and the presence of fungal infection on garlic storage; (2) evaluate the changes in the qualitative properties of garlic during the storage period and based on different levels of applied treatments; and (3) monitor garlic aroma changes under different storage conditions using the E-Nose. The novelty of this research was the utilization of E-Nose as a non-destructive method in estimating the correlation of changes in the qualitative properties related to garlic and its aroma alteration based on different storage conditions. According to this solution, some significant features could be monitored in real-time such as product quality alteration; the effectiveness of the storage parameters; the capability of packaging as well as its Inhibitory properties; and the efficiency of MAP. Also, this solution can be an introduction to the industrialized enhancement of future storage systems.

## 2 Materials and methods

### 2.1 Experimental setup

Samples were supplied directly from a garlic farm (Hamedan, Iran). The garlic cloves were immune to any damage, decay, and stain. Subsequently, the pathogenic inoculations were made in the plant pathology laboratory, Department of Plant Protection, Faculty of Agriculture, Bu-Ali Sina University, Hamedan, Iran. To sterilize the surface of cloves, 75 % ethanol was used [18]. The inoculations were done by three pathogenic fungi, namely *Fusarium oxysporum f. sp. Cepae* (FF), *Alternaria embellisia* (AF), and *Botrytis allii* (BF). Potato dextrose agar was used to grow fungi at 80 % relative humidity (RH) and a temperature of 22 °C. In addition, the incubation process was carried out for 168 h before inoculation [19]. The cloves were immersed in spore suspension of fungal pathogens (1 × 10^6^ spores/mL) for 30 s. Besides, controlled samples (CT) were immersed for 30 s in sterile distilled water only.

### 2.2 MAP

After inoculation, samples were placed in packages with two materials i.e. low-density polyethylene packaging (LP) and silicone Nano-emulsions packaging (SP). The purpose of using two materials in the packaging was the different effectiveness in the degradation procedure of stored food [20]. The differences in inhibitory properties of packaging and their protective characteristics cause diverse intensities in the growth of microorganisms associated with food spoilage. After placing the samples inside the packages, a modified atmosphere with a composition of 1 % oxygen (O_2_), 5 % carbon dioxide (CO_2_), and 94 % nitrogen (N_2_) were also injected into half of the packages, immediately [21]. Finally, the packages were sealed and stored at ambient conditions (22 ± 1 °C and 30–45 % RH) for 28 days. Each TC was inspected every four days. Each packaging contained ten pieces of garlic cloves weighing approximately 50 ± 5 g. According to the different levels of applied treatments, 16 TCs were obtained, and 13 samples were prepared for each of the TC. Noteworthy, 3 samples of each TC were considered for qualitative properties evaluation and 10 samples were prepared for olfactometry tests. Therefore, the total number of samples was 208.

### 2.3 Qualitative properties

#### 2.3.1 Weight reduction (WR)

To estimate the WR, the weight of the samples immediately after packing was considered the initial weight (W_i_). An analytical balance (GF-600, ±0.001 g, max 610 g, AND, Japan) was used to measure the weight of the samples (W_t_). The WR was computed as follows:

(1) $$\text{WR}=\left[\left(W_{i}-W_{t}\right)/W_{i}\right]\times 100$$

#### 2.3.2 Browning index (BI)

To quantify the BI, a portable digital colorimeter (hp-200, Shenzhen Handsome Technology Co., Ltd., China) was used. To eliminate the destructive environmental influences on the colorimetric experiments, the tests were conducted under the same environmental circumstances. The CIE L∗a∗b∗ coordinates were detailed, and the BI was calculated using Eq. (2) and Eq. (3) [22].

(2) $$x=\left(a^{*}+1.75L^{*}\right)\;/\;\left(5.645L^{*}+a^{*}-3.012b^{*}\right)$$

(Eq.3) BI=100(x−0.31)/0.172

#### 2.3.3 Acidity (pH)

To calculate pH, garlic cloves belonging to each TC were grated. To achieve a homogenized extract, it was then grounded, and a 40 μm filter paper was used to filter the extract. The pH was assessed by a pH meter (pHS-BW, Bante, Italy) with a resolution of 0.01.

#### 2.3.4 Total soluble solids (TSS)

Based on the manner presented by Gholami et al., TSS was quantified at 28 ± 1 °C utilizing a refractometer (PAL-1, Tokyo, Japan) with a resolution of 0.01 [23]. In TSS experiments which were conducted in three replications, the extract of garlic was dripped on the prism.

#### 2.3.5 Tissue temperature (TT)

The TT of cloves was captured by an infrared thermograph system (E40, FLIR, Italy). This system features a resolution of 120 × 160 pixels; a frame rate of 60 Hz; a field of view of 19° × 25°; a thermal sensitivity of <0.07 °C at 30 °C; and a spectral range of 7.5–13 μm. To eliminate the adverse influence of environmental circumstances, the images were captured under similar conditions. Thermal images were always captured at 30 ± 1 °C in six replications.

#### 2.3.6 Toughness (TN)

The TN defines the intrinsic resistance of tissue to the rupture [24]. The area under the stress-strain curve to the rupture point and in the elastic limitation represents the TN [25]. Puncture tests were directed on cloves utilizing a deformation analysis dynamometer (Bbt1-Fro.5th.D14, Zwick/Roell, Ulm, Germany) supplied by a 500 N load cell (X Force Hp, Zwick/Roell, Ulm, Germany) and a probe with 3 mm in diameter. The probe penetrated 10 % of the sample diameter at a speed of 20 mm/min.

#### 2.3.7 Headspace composition (HC)

To investigate the HC changes, three samples related to each TC were kept separately in the same storage conditions. The changes in O_2_ and CO_2_ concentration were monitored by a gas analyzer (Witt-Gastechnik GmbH, V6i, oxybaby, Germany).

### 2.4 Garlic aroma evaluation

**Fig. 1:**
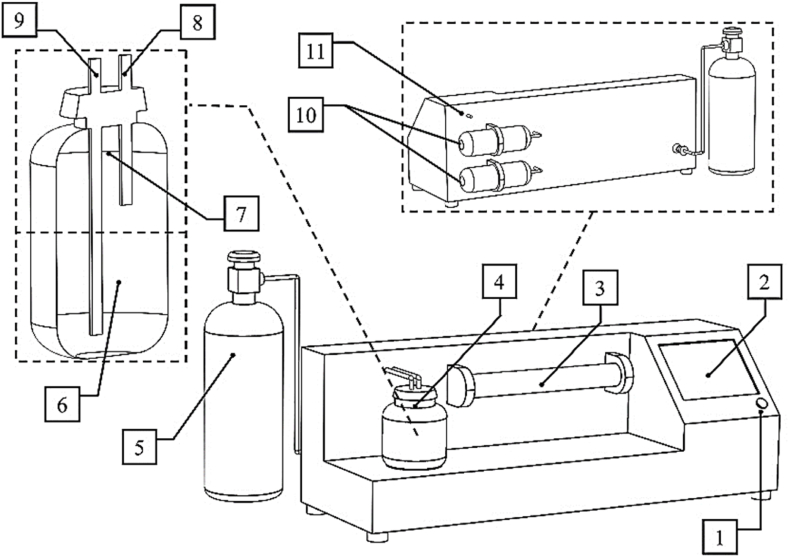
The utilized E-Nose: 1. Power button, 2. Touch screen, 3. Sensors chamber, 4. Sample container, 5. Oxygen gas tank, 6. Sample location, 7. Headspace, 8. Inlet path from the carbon filter, 9. Output path to the sensors, 10. Carbon filters, 11. Suction pump output.

The fabricated E-Nose (Fig. 1) has been fully described in previous research [14]. Nine metal oxide semiconductor (MOS) sensors (Hanwei Electronics Co., Ltd., Henan, China) were embedded in the sensors chamber as an array. Chemical sensors employed in E-Nose must be well sensitive to VOCs to detect different types of aromas. The specifications of the utilized sensors are presented in Table 1.

**Table 1:** MOS sensors specification used in designed E-nose based on revealed datasheets.

Name	Sensitivity Applications	Detection Ranges (ppm)
MQ-2	LPG, Smoke, Alcohol, Propane, Hydrogen, Methane and Carbon Monoxide	200 to 10000
MQ-3	Alcohol	25 to 500
MQ-4	CH_4_, Natural gas	200 to 10000
MQ-5	Town gas, Natural gas, Iso-butane, Propane, LPG, LNG	200 to 10000
MQ-6	LPG, LNG, Iso-butane, Propane	200 to 10000
MQ-7	Carbon monoxide	20 to 2000
MQ-8	Hydrogen (H_2_)	100 to 10000
MQ-9	CO, Combustible gas	500 to 10000 (CH_4,_ LPG) 20 to 2000 (CO)
MQ-135	Ammonia (NH_3_), Benzene, Alcohol	10 to 300 (NH_3,_ Alcohol) 10 to 1000 (Benzene)

The E-Nose experiments included baseline adjustment, headspace insertion, and sensor retrieval phases. In the baseline adjustment and sensor retrieval phases, the sensor recuperation operations were performed to restore the response of the sensors to the reference point and eliminate any inaccuracies in sensor duties. While the headspace insertion accrued, the sensors generated electrical signals as responses. The best time length in each phase was selected experimentally as 60, 40, and 60 s for the baseline adjustment, headspace insertion, and sensor retrieval phases in the order of appearance. These durations resulted in the most fitting response and sensor preparation for the following cycles. Noteworthy, the main function of baseline adjustment and sensor retrieval phases was to increase the stability in the sensors. To minimize the influences of temperature and humidity on the stability in the sensors’ performance, the olfactometry tests were carried out in a certain place and at the same time of the day. To enhance the precision of analyses, the olfactory investigation was performed in 10 replications.

### 2.5 Statistical analysis

To investigate the influence of studied treatments viz. packaging atmosphere, packaging material, and fungal infection on the qualitative properties of garlic cloves during storage, the ANOVA was made by the factorial test in a completely randomized design. Additionally, the comparison of the means was conducted based on the Duncan method. These analyses were performed in SAS v9.4 (SAS Inst. Inc., Cary, NC).

The data acquired by the E-Nose were evaluated by diverse approaches. The multivariate analysis was employed utilizing the PCA. PCA is a scaling-down approach that operates to simplify information of the outsized datasets. The LDA and BPNN approaches were employed to categorize the diverse classes of aromas associated with different TCs. The LDA gathers samples associated with the same category closer, while it scatters samples related to dissimilar categories. The BPNN aims to decode the issues according to the rules that the human brain noted. To evaluate the correlation between the aroma's profiles and qualitative properties, the PLSR was applied. PLSR, PCA, and LDA were implemented by the unscrambler X 10.4 (CAMO ASA, Norway), while BPNN was conducted using MATLAB R2015b (The Mathworks Inc., Natick, MA, USA).

## 3 Results and discussions

### 3.1 Qualitative properties

#### 3.1.1 WR & BI

**Fig. 2:**
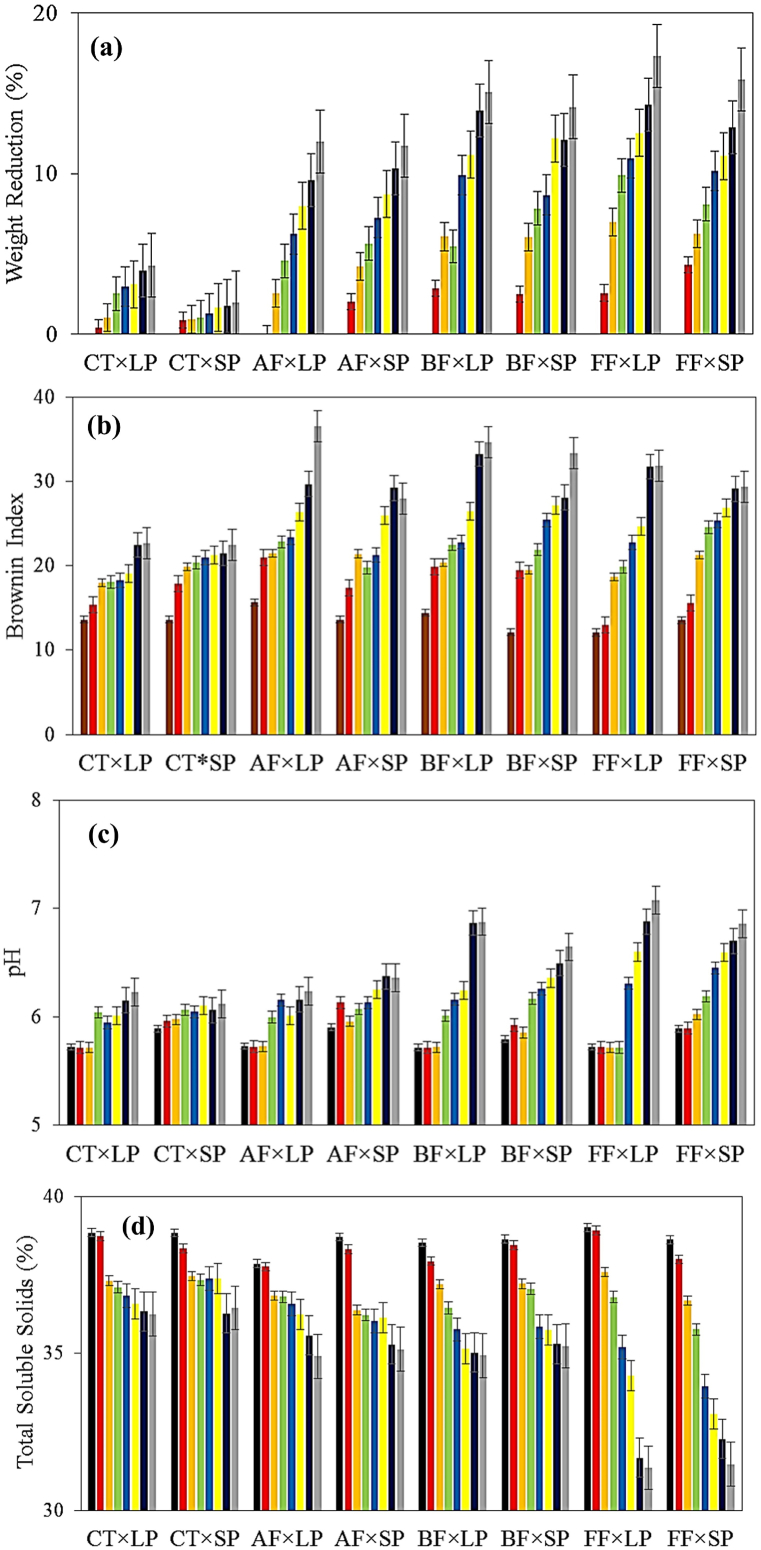
Physical qualitative properties related to different combinations of fungal infection × packaging material treatments in ■ Day0,  Day4,  Day8,  Day12,  Day16,  Day20,  Day24, and  Day28: (a) WR, (b) BI, (c) pH, and (d) TSS.

During the storage period, WR and BI increased in all samples. The spoilage due to fungal infection destroyed the structure of the tissues more rapidly and the moisture content was diffused with more flux. Therefore, the fungal contamination caused the changes in WR and BI to intensify. The highest WR was observed in the order of appearance of BF-, FF-, and AF-infected samples, while the highest BI was detected in BF-, AF-, and FF-infected samples, respectively. The use of SP packaging and MAP reduced the rate of variation in WR and BI indices. The reason for this point was the better isolation of the samples from the outside environment by the packing of the SP, as well as the low respiration rate created by the modified atmosphere (Fig. 2).

ANOVA results revealed that all effects of the studied treatments on changes in the WR and BI were significant at the level of 1 %. Besides, modeling was performed with a correlation coefficient of 0.99 and 0.92 accounted for WR and BI, respectively (Table 2). The comparison of the means demonstrated that the highest and lowest WR was observed in the TCs of “FF × LP × Day28” (17.33 %) and “CT × SP × Day4” (0.86 %), respectively. Moreover, the lowest and highest BI was monitored in TCs of “FF × LP × Day4” (12.07) and “AF × LP × Day20” (36.53), respectively. Riudavets et al.*,* inspected the effect of MAP on *aflatoxin* contamination in maize [26]. It was declared that the MAP and storage duration influenced the rate of WR. Gholami et al., investigated the effect of MAP and Nano-packaging to improve the storage conditions of mushrooms [23]. The results showed that the MAP had a significant impact on the qualitative properties as well as the respiration rate of mushrooms.

**Table 2:** The ANOVA of the studied treatments on the qualitative properties of garlic cloves.

sources	df	WR	BI	pH	TSS	TT	TN	O_2_	CO_2_
a^a^	3	1053.7^b^	658.8^b^	2.3^b^	103.2^b^	171.3^b^	2130^a^	98.3^b^	95.2^b^
b^b^	1	93.5^b^	116.4^b^	0.4^b^	0.08^a^	7.9^a^	1295.1^b^	189.9^b^	50.9^b^
c^c^	1	2064.6^b^	4463.6^b^	14.3^b^	398.6^b^	1516.9^b^	14000.9^b^	80.1^b^	264.3^b^
a × b	3	258.5^b^	129.0^b^	0.1^b^	6.1^b^	15.1^ns^	1122.5 ^ns^	17.4^b^	23.7^b^
a × c	3	400^b^	760.7^b^	3.7^b^	105.6^b^	115.5^b^	2720.2 ^ns^	45.5^b^	32.1^b^
b × c	1	106.7^b^	104.6^b^	0.8^b^	1.6^b^	17.5 ^ns^	463.7 ^ns^	126.7^b^	46.0^b^
a × b × c	3	146.9^b^	301.1^b^	0.5^b^	8.03^b^	60.9 ^ns^	2260.4 ^ns^	10.4^b^	14.1^b^
Error	126	47.9	538.8	0.02	1.6	606.4	83456.7	13.4	0.06

Abbreviations: WR, Weight Reduction; BI, Browning Index; TSS, Total soluble solids; TT, tissue temperature; TN, Toughness.

a Fungal infection treatment.

b Packaging atmosphere.

c Packaging material.

#### 3.1.2 pH & TSS

During the storage period, pH and TSS faced an increasing and decreasing trend, respectively (Fig. 2). The fungal infection intensified the changes in pH and TSS indices. The presence of fungal pathogens which are acidic caused the cloves to be water-soaked. Hence, pathogenic progression led to an increase in pH. This consequence is in agreement with the results of others [[27], [28], [29], [30]]. As mentioned earlier, fungal infection causes a genetic abnormality of garlic such as the waxy breakdown. Here, fungal infection caused a decrease in the TSS through the structural changes of garlic as well as the transformation of its tissue into a transparent state. Noteworthy, the utilization of SP packaging and MAP limited the pH and TSS alteration.

ANOVA results revealed that all effects of the studied treatments on changes in pH and TSS indices were significant. The models were conducted with a correlation coefficient of 0.99 (Table 2). The comparison of the means related to the pH values, showed that the TCs of “CT × LP × Day4” and “FF × LP × Day28” had the lowest (5.71) and highest (7.07) mean values, respectively. Moreover, the highest and lowest mean values of TSS were observed in the TCs of “CT × LP × Day4” (39.01 %) and “FF × LP × Day28” (31.35 %), respectively. Consistent with these results, several studies have evaluated the effect of fungal infection, packaging material, and MAP treatments on changes in pH and TSS indices [31,32].

#### 3.1.3 TT

**Fig. 3:**
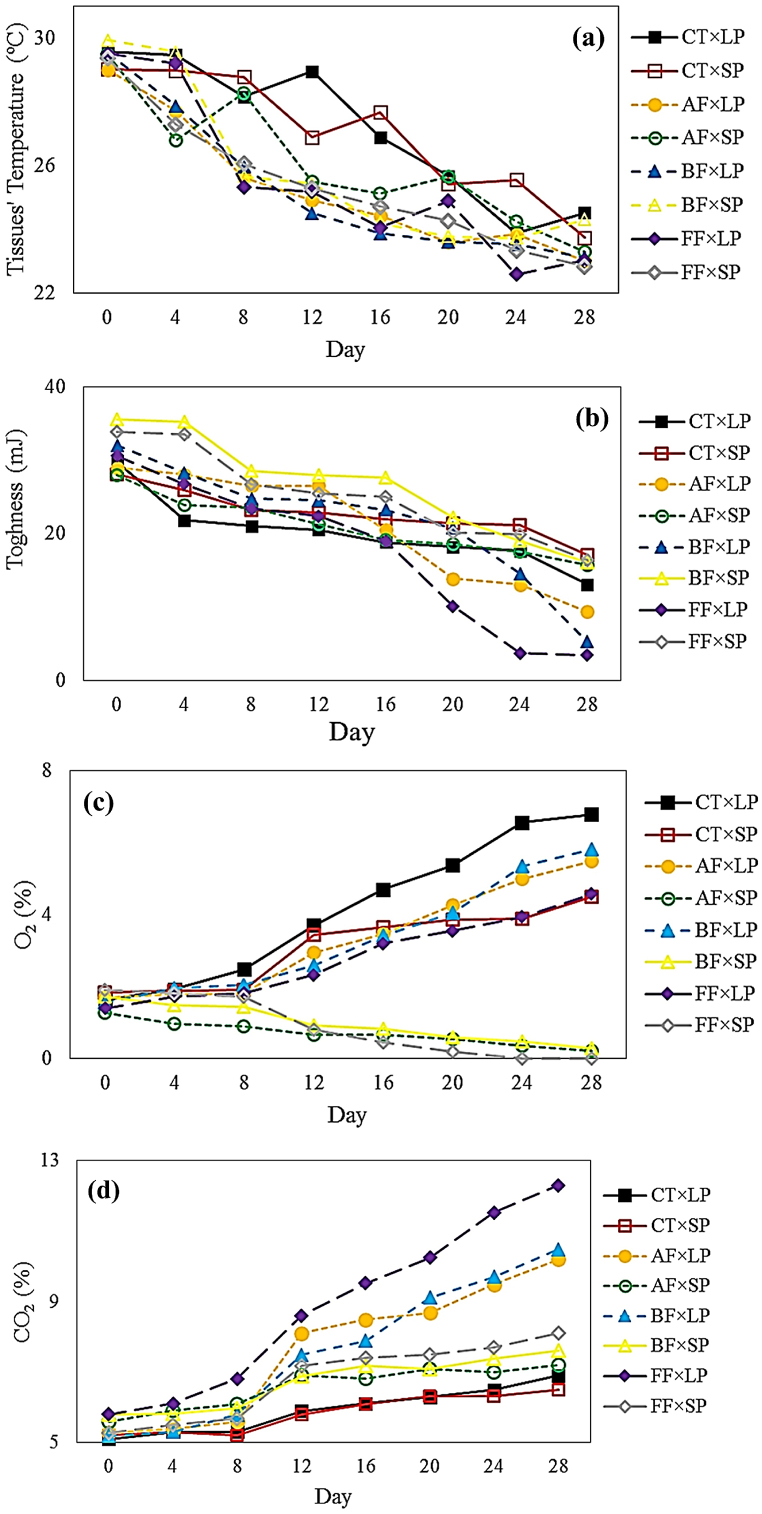
Chemical qualitative properties related to different combinations of fungal infection × packaging material treatments: (a) TT, (b) TN, (c) O_2_ Concentration, and (d) CO_2_ Concentration.

The results of TT capturing by the thermal camera revealed that the TT decreased over the storage period. As expected, the progress of product respiration, tissue degradation, and promotion of garlic metabolism were the reasons for the TT decreasing. The spread of decay caused by the fungal infection increased the volume of damaged and dead tissue. Therefore, the TT index decreased more strongly in the presence of fungal infection. The highest decrease in TT was observed in FF-, BF-, and AF-infected samples, respectively. Owing to the simultaneous utilization of SP packaging as well as the MAP, better quality retention occurred (Fig. 3).

The ANOVA revealed that all the simple effects of the studied treatments on the TT alteration were significant, while only the interaction effect of “fungal infection × packaging material” was significant over the storage period (Table 2). The comparison of the means related to the TT values associated with packaging material treatment testified that the TT values related to the samples packed in SP were higher than those kept in LP. Moreover, the means comparison exposed that the TCs of “FF × Day28” and “BF × Day0” recorded the lowest (22.92 °C) and highest (29.74 °C) mean values of TT, respectively. Paulsen et al.*,* declared that the TT is firmly influenced by the respiration of tissue and its structure [33]. It was also reported that the packaging with high inhibitory characteristics could govern better the destructive traits and quality retention.

#### 3.1.4 TN

The energy required for the rupture point (TN) decreased over the storage period which is caused by the destruction of the tissue's structure (Fig. 3). Also, the role of fungal pathogens intensified this decreasing trend in the TN index. The MAP controlled garlic respiration, which led to a decrease in tissue destruction rates. Hence, the MAP employment prevented the TN reduction. The results depicted that the slightest changes in TN were related to the TC of “CT × SP” (11 mJ), while the most changes were observed in the TC of “FF × LP” (27.20 mJ).

ANOVA revealed that despite the simple effects of the studied treatments on TN changes which were significant, the interactions were insignificant (Table 2). The comparison of the means related to the simple effect of fungal infection treatment testified that the highest mean of TN belonged to the BF-infected samples. At the same time, there was no significant difference between other levels. Also, the comparison of the means associated with the simple effects of the packaging material as well as packaging atmosphere treatments exposed that the TN gained the highest values in the samples packed in SP with the modified atmosphere. Barikloo and Ahmadi, used novel packaging to maintain the quality of strawberries as well as to enhance appropriate storage conditions [34]. The results showed that the packaging and storage period treatments were significant in qualitative properties.

#### 3.1.5 HC

Respiration is the process by which plants receive O_2_ to break down the carbohydrates in the plant into CO_2_ and water. Although the concentration of O_2_ had to be reduced in packaging due to the presence of respiration, the concentration increased on the contrary. The reason for this incident was the unavoidable penetration of air from outside the package into it. This penetration was to such an extent that it exceeded even the amount required for respiration of the uninfected garlic and caused an increase in O_2_ concentration. The penetration of the ambient atmosphere was conducted with much less flux in SP packaging than that of LP. Hence, the rate of increase of O_2_ concentration in SP packaging was much lower than that of LP. The heterogeneity of O_2_ changes in both types of packages was due to the different inhibitory properties. It should be noted that the O_2_ concentration in the SP packaging of infected samples decreased. It can be inferred the reason for this was that as fungal infection progressed, the respiration of garlic increased to such an extent that the consumption of O_2_ was higher than the total concentration of O_2_ in packaging and the penetration rate. Notably, the concentration of O_2_ in SP packaging which contained FF-infected samples, was zero from Day24 (Fig. 3).

The concentration of CO_2_ increased over the storage period, especially its changes jumped from Day12 onwards (Fig. 3). Although the MAP minimized respiration, one of the factors that increased the concentration of CO_2_ in the packaging was the metabolism of garlic. Pathogenic infection increased the metabolism of samples, so the increasing rate of CO_2_ in the packages containing the infected samples was higher than in the CTs. As in the last of the storage period, the lowest O_2_ concentration as well as the highest CO_2_ concentration, were measured in packaging containing FF-, BF-, and AF-infected samples in the order of appearance.

ANOVA revealed that all effects of the studied treatments on the changes in O_2_ and CO_2_ were significant at the level of 1 %. Also, modeling was conducted to describe the O_2_ and CO_2_ changes with a coefficient of determination of 0.98 and 0.99, respectively. The comparison of the means of the studied treatments testified that the lowest and highest O_2_ concentrations belonged to the TCs of “FF × SP × Day28” (0 %) and “CT × LP × Day28” (6.78 %), respectively. In contrast, the lowest and highest CO_2_ concentrations were observed in the TCs of “CT × LP × Day0” (5.1 %) and “FF × LP × Day28” (12.29 %), respectively.

### 3.2 Garlic aroma evaluation

#### 3.2.1 Array's responses

The estimation of the quality degradation of samples was done by visual inspection. Moreover, the tissue destruction percentage of all the samples was calculated. Complete instructions for this procedure are provided in the previous study [14]. Although, the effects of inoculation had not progressed to such an extent that tissue destruction was observed on Day0, the elongation of the storage period resulted in tissue destruction by the fungal contamination. The percentage of tissue destruction of the CT samples was 0 %, 1.25 %, and 13.13 %, on Day4, Day8, and Day12, respectively. Furthermore, tissue destruction of the samples infected with various fungi on Day4, Day8, and Day12 were approximately 11.87 %, 30 %, and 42 %., respectively. The tissue destruction in the CT samples and related to Day16, Day20, Day24, and Day28 were 23.13 %, 39.33 %, 41.25 %, and 56.25 %, respectively. From Day16 to Day28, the percentage of tissue destruction of infected samples was 58.13 %, 97.5 %, 100 %, and 100 %, respectively.

**Fig. 4:**
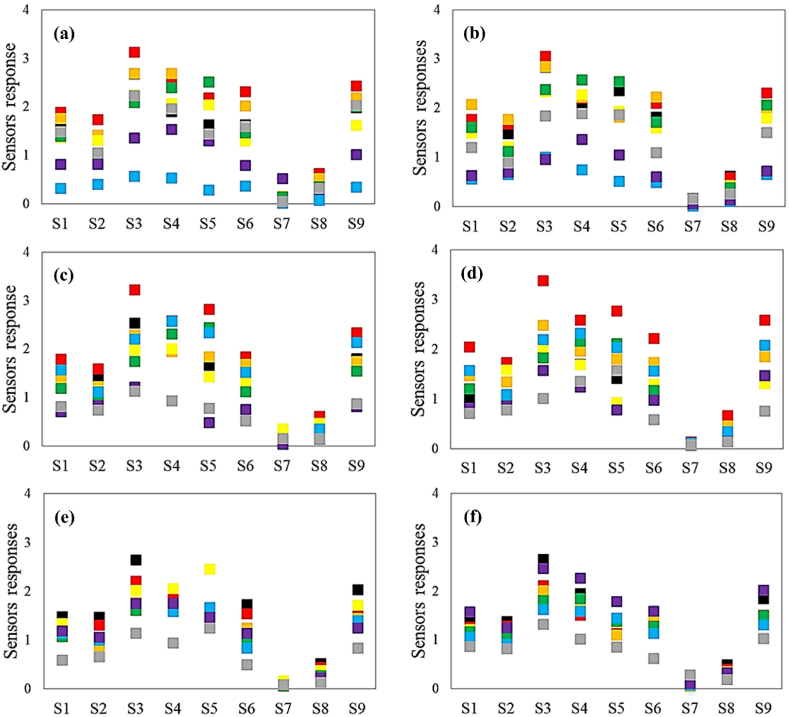
The array's responses toward the aromas of treatment combinations in ■ Day0,  Day4,  Day8,  Day12, Day16,  Day20,  Day24, and  Day28: (a) CT × LP in ambient air, (b) CT × SP in ambient air, (c) BF × LP in ambient air, (d) BF × SP in ambient air, (e) BF × LP in MAP, and (f) BF × SP in MAP.

The set of sensors' responses toward the aroma of different TCs had a unique pattern, while the sensors’ responses did not expose an exclusive trend (Fig. 4). The utilization of different packaging materials also affected the stimulation rate of the sensor array. The SP packaging utilization resulted in more stimulation of all sensors against the aroma of infected samples than CT. This result was due to pathogenic contamination, which caused more decay of garlic and ultimately more intense diffusion of aroma. The use of different levels of packaging atmosphere treatment was also associated with changes in the response of the sensors. Using the MAP due to the quality retention of garlic was responsible for the excitation of the sensors at lower levels. The use of ambient atmosphere in packaging resulted in greater crop spoilage and thus different stimulation. Shen et al., inspected the fungal infection levels by the E-Nose [35]. The peanuts were artificially inoculated with five *Aspergillus* strains and stored for nine days. The E-Nose responses were distinctive at different storage stages. The results revealed that the E-Nose approach facilitates the early detection of fungi.

#### 3.2.2 PCA results

**Fig. 5:**
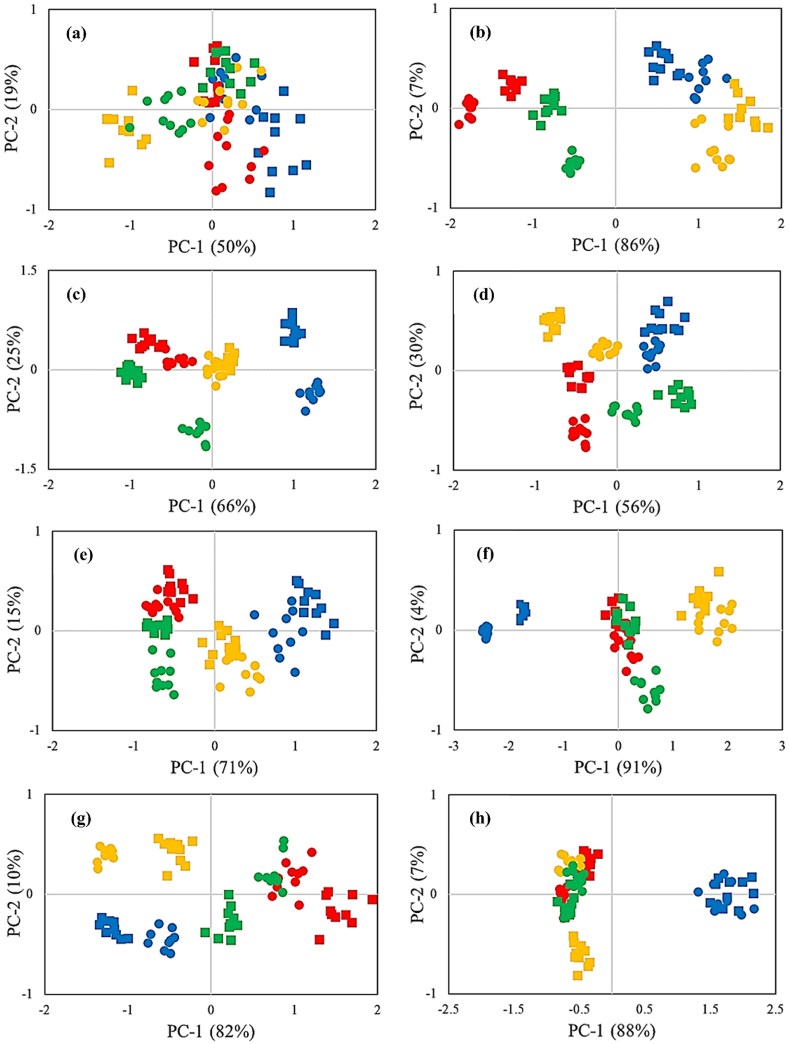
PCA results for the data obtained by the E-Nose related to different TCs in (a) Day0, (b) Day4, (c) Day8, (d) Day12, (e) Day16, (f) Day20, (g) Day24, and (h) Day28;  CT × LP,  CT × SP,  AF × LP,  AF × SP,  BF × LP,  BF × SP,  FF × LP,  FF × SP.

The PCA consequences (Fig. 5) demonstrated the first and second PCs included 19 % and 50 % input variables on Day0, respectively. On Day0, the patterns related to different TCs overlapped, and the discrimination of the effects was not provided. It was expected since the potential effects of the employed treatments were not sufficient to be observed. The sensors S8, S3, and S6 had the highest loadings in patterning scores in the first principal component (PC-1) direction, respectively. Also, the sensors S5, S7, and S9 had the highest loadings in the patterning of scores in the second principal component (PC-2) order.

Notably, 93 % of the total variance of input variables was included on Day4. There was a slight overlap between the patterns of the CT and BF-infected samples. The scores related to CT and BF-infected samples occupied the positive side of the PC-1 axis, whereas the score values of FF and AF-infected samples were arranged on the opposing side. The E-Nose had a satisfactory reaction against the presence of infection but could not fully detect the levels of infections. Moreover, the SP packaging utilization also increased the score values in the PC-2 direction.

The sensors S8, S9, S1, S3, S5, and S4 in the order of appearance had the highest loadings in the patterning of scores in the PC-1 direction on Day4. Also, the sensors S7, S5, and S2 exposed the most impact on the scores patterning in the PC-2 direction, respectively.

On Day8 and Day12, the score patterns related to different TCs were apart. During this period, the dispersion within all categories condensed over storage, while the distance between patterns increased. From Day16 onwards, the distances between the categories related to the TCs were decreased, so the overlap between different levels of infection treatment began. The reason for this was that the decay caused by fungal contamination had reached such an extent that a significant amount of tissue had been destroyed, and the aroma did not undergo noticeable changes based on the presence of different fungi. From Day16 onwards, the decay of garlic reached such a level that it overshadowed the effect of different levels of packaging material treatments. Although the score values related to the aroma of the infected samples overlapped upon completion of the storage period (Day28), the CT samples had a discrete pattern on the positive side of the PC-1 axis.

The PCA results reveal that the trend of aroma alteration in the samples kept under MAP conditions was the same, with the difference that as the decay of garlic was controlled and the scattering of the score decreased. Therefore, using the MAP approach resulted in a forward time shift. This time shift was such that the patterns related to the different levels of fungal infection treatment were fully separated from Day12 to Day24, and the overlap started from Day28.

Consistent with these results, Cui et al., developed an E-Nose to inspect infestation in tomatoes [36]. The results indicated that the E-Nose could accurately discriminate the infested plants as well as mechanically damaged plants. They noted that changes in storage conditions can affect the changes in tomato aroma.

#### 3.2.3 Classifiers results

PCA results revealed that implementing different TCs in diverse storage stages of garlic influenced its aroma. Subsequently, the classifiers were applied to categorize the aroma based on the differences and similarities. Equal priorities were used for all applied factors in the running of the LDA. In the required settings before the implementation of the BPNN method, the optimal number of hidden layer neurons was selected 14 by trial-and-error. The reason for this was the complexity of the matrix of TCs. Therefore, the topology for the classification based on fungal infection was designed 9-14-4, while it was 9-14-2 for the classification based on packaging atmosphere and packaging material treatments. The data used in BPNN were randomly arranged in three sets, namely the training, validation, and test sets which accounted for 70 %, 15 %, and 15 % of the data, respectively.

On Day0, none of the classifiers succeeded in complete classification based on different levels of fungal infection treatment (Table 3). The reason for this was that the effect of the fungal infection treatment was not enough to affect the aroma on Day0. From Day4 to Day20, the different levels of infection treatment were fully classified by the LDA and BPNN methods. From Day24 onwards, misclassification was seen in the LDA approach. Finally, the performance of both approaches was associated with errors on Day28. The reason for this was that after a while when the corruption caused by various fungal infections completely affected the garlic tissue, it was only possible to comment on infection not the type of fungus.

**Table 3:** The accuracies of LDA and BPNN classification based on different studied treatments.

	Fungal Infection		Packaging Material		Packaging Atmosphere	
	LDA (%)	BPNN (%)	LDA (%)	BPNN (%)	LDA (%)	BPNN (%)
Day0	51.25	38.8	75	82.5	75	82.5
Day4	100	100	83.75	92.5	81.25	82.5
Day8	100	100	81.25	97.5	83.75	90
Day12	100	100	81.25	96.3	100	100
Day16	100	100	73.75	82.5	100	100
Day20	100	100	90	97.5	100	100
Day24	98.75	100	95	96.3	100	100
Day28	8.75	87.5	93.75	92.5	95	96.3

In categorizing the aroma based on the packaging materials treatment, the LDA and BPNN approaches did not succeed in complete classification in any of the storage stages. The point that strongly stood out was that the classification accuracy increased with the progression of storage periods from Day0 to Day28 stages. This meant that the effect of packaging material treatment was shown in the long run and the quality of the product could be maintained more by controlling the amount of air exchange with the outside environment.

The classification of aroma based on different levels of packaging atmosphere treatment exposed that from Day0 to Day8 none of the LDA and BPNN were able to categorize correctly. The control of the garlic respiration by the MAP caused the garlic quality associated with Day8 to be the same as Day0 as much as possible. From Day12 to Day20, the aromas were fully classified according to the packaging atmosphere treatment, while the misclassifications occurred in the performance of both approaches on Day28. As with the results of PCA, it was found that if the packaging atmosphere is modified at best, it can significantly improve the preservation of product quality. Besides, MAP technology can suppress the effect of other perilous factors by controlling respiration. The point that was highly valued is that the effect of packaging atmosphere treatment on the aroma changes was greater than packaging material treatment, while the presence of packaging with high sealing properties can multiply the efficiency of packaging atmosphere treatment.

### 3.3 PLSR results

To correlate between the variables by PLSR, the array's response and the results of qualitative experiments were considered the input and output of the model, respectively. The modeling of regression related to the qualitative properties was conducted by Eq. (4). The Y_Characteristic_ defined the predicted values. The coefficient of A_0_ was constant. Also, the coefficients of A_1_ to A_9_ belonged to the predictor variables (response of the sensors).

(4) $$Y_{\text{Characteristic}}=A_{0}+A_{1}\;\left(S1\right)+A_{2}\;\left(S2\right)+A_{3}\;\left(S3\right)+A_{4}\;\left(S4\right)+A_{5}\;\left(S5\right)+A_{6}\;\left(S6\right)+A_{7}\;\left(S7\right)+A_{8}\;\left(S8\right)+A_{9}\;\left(S9\right)$$

The PLSR results (Table 4) revealed that the performance of WR modeling was acceptable (R^2^ = 0.96). In predicting the WR, the sensors S8, S7, S2, and S6 had the highest loadings in the correlation, respectively. Also, the model of the BI index according to the aroma profiles exposed a satisfactory performance (R^2^ = 0.86). The sensors S8, S5, S1, and S6 had the highest loadings in the interpretation of the BI trend, respectively. The results of the correlation between the changes in pH and the aroma of garlic revealed that 87 % of pH changes were correlated to the changes in sensor responses. Notably, the sensors S7, S2, and S8 had the highest loadings in the pH prediction. The performance of the PLSR model demonstrated that about 92 % of the array responses were interconnected by the TSS differentiations. During storage, changes in TSS had the highest correlation with changes in the response of the sensors S7 and S5, respectively.

**Table 4:** Regression coefficients estimated by the model obtained from the PLSR.

Qualitative Properties	A_0_	A_1_	A_2_	A_3_	A_4_	A_5_	A_6_	A_7_	A_8_	A_9_	R^2^
WR	2.79	−1.71	−8.48	−2.26	1.19	0.54	8.00	9.02	20.61	0.53	0.96
BI	21.79	−9.86	3.51	−0.13	−5.27	15.46	−7.08	−5.22	20.85	0.29	0.86
pH	5.55	0.33	1.08	0.22	−0.03	−0.22	−0.42	1.18	−0.51	−0.08	0.87
TSS	35.38	0.89	−0.24	0.43	−0.28	1.34	−0.14	−9.60	0.68	−0.72	0.92
TT	18.33	−1.36	5.71	−1.42	1.06	−1.12	1.65	24.32	5.48	−0.62	0.82
T	19.58	−1.64	−1.97	−1.97	2.72	7.06	−2.95	5.19	−7.55	−3.96	0.24
O_2_	18.88	10.26	−10.41	−9.77	0.05	−8.23	10.09	−21.20	11.02	−0.99	0.89
CO_2_	6.49	−3.70	1.12	−1.57	2.51	2.30	−0.70	1.65	−7.02	0.89	0.97

Abbreviations: WR, Weight Reduction; BI, Browning Index; TSS, Total soluble solids; TT, tissue temperature; TN, Toughness.

By modeling the correlation between the changes in TT and the array responses, the prediction of the independent variable was determined to be acceptable (R^2^ = 0.82). To describe the TT changes, the sensors S7, S2, and S8 had the highest loadings, respectively. The PLSR results showed an unpleasant performance in TN modeling. Only 24 % of changes in array responses were correlated to the TN alteration. The performance of the PLSR in modeling the O_2_ during storage was 89 %. The sensors S7, S8, S2, S1, and S6 had the highest loadings in determining the correlation between the changes in the O_2_ and the response of the array. The results of the CO_2_ regression revealed that 97 % of its differences were linked to the changes in sensor responses. Notably, the sensors S8, S1, S4, and S5 had the highest loadings in the CO_2_ predicting.

In general, these results proved that the changes in the aroma of garlic had a significant correlation with the changes in qualitative properties. Among the qualitative properties, the physical and mechanical traits had the highest and lowest correlation with aroma changes, respectively. As mentioned earlier, the smallest changes in the structure and tissue of garlic cause dramatic changes in its aroma. Therefore, these results presented those changes in garlic aroma are related to changes in its physical, chemical, and even thermal traits.

## 4 Conclusion

Treatments such as packaging material, packaging atmosphere, and fungal infection were used to investigate the qualitative properties of garlic during storage. The WR, BI, and pH indices increased during the storage period, while the TSS, TT, and TN traits decreased. The fungal infection intensified these trends, whilst the MAP as well as the SP packaging reduced the greatness of the changes. The ANOVA results showed that, except for TT and TN traits, all effects of the studied treatments had a significant effect on the changes related to the rest of the indices. The PCA clarified that during the storage period, the sensitivity of the E-Nose toward the fungal contamination had an increasing-decreasing trend. Also, the PCA results proved the usefulness of using MAP and optimal packages with high-insulating properties. The outcomes of LDA and BPNN were consistent with the results presented by the PCA method. The PLSR results proved that the changes in the aroma of garlic had a significant correlation with the changes in qualitative properties. According to the results of this research, it was concluded that the E-Nose can be used as a complementary strategy in non-destructive monitoring of effective conditions in product storage as well as to expand their quality control.

## CRediT authorship contribution statement

**Alireza Makarichian:** Writing – original draft, Software, Resources, Methodology, Data curation. **Ebrahim Ahmadi:** Writing – review & editing, Validation, Supervision, Software, Methodology. **Reza Amiri Chayjan:** Validation, Supervision, Methodology, Investigation. **Doostmorad Zafari:** Validation, Methodology, Investigation. **Seyed Saeid Mohtasebi:** Validation, Methodology, Investigation, Data curation.

## Informed consent

Not require.

## Ethical review

This study does not involve any human or animal testing.

## Data availability statement

The data that support the findings of this study are available from the corresponding author upon reasonable request.

## Declaration of competing interest

All authors declare that there is no conflict of interest.

## References

[1] Takagi H. (2020).

[2] Hayat S., Ahmad A., Ahmad H., Hayat K., Khan M.A., Runan T. (2022). Garlic, from medicinal herb to possible plant bioprotectant: a review. Sci. Hortic..

[3] Gasser K., Sulyok M., Spangl B., Krska R., Steinkellner S., Hage-Ahmed K. (2023). Fusarium proliferatum secondary metabolite profile in vitro depends on the origin of the isolates and is clearly reduced in stored garlic. Postharvest Biol. Technol..

[4] Madhu B., Mudgal V.D., Champawat P.S. (2019). Storage of garlic bulbs (Allium sativum L.): a review. J. Food Process. Eng..

[5] Rahman M.S. (2007). Handbook of food preservation.

[6] Ncube L.K., Ude A.U., Ogunmuyiwa E.N., Zulkifli R., Beas I.N. (2020). Environmental impact of food packaging materials: a review of contemporary development from conventional plastics to polylactic acid based materials. Materials.

[7] Olveira-Bouzas V., Pita-Calvo C., Vázquez-Odériz M.L., Romero-Rodríguez M. (2021). Á., Evaluation of a modified atmosphere packaging system in pallets to extend the shelf-life of the stored tomato at cooling temperature. Food Chem..

[8] Jafarzadeh S., Nafchi A.M., Salehabadi A., Oladzad-Abbasabadi N., Jafari S.M. (2021). Application of bio-nanocomposite films and edible coatings for extending the shelf life of fresh fruits and vegetables. Adv. Colloid Interface Sci..

[9] Ferioli F., Giambanelli E., D'Antuono L.F. (2022). Non-volatile cysteine sulphoxides and volatile organosulphur compounds in cloves of garlic (Allium sativum L.) and elephant garlic (Allium ampeloprasum L.) local accessions from northern and central Italy. J. Sci. Food Agric..

[10] Nakagawa K., Maeda H., Yamaya Y., Tonosaki Y. (2020). Maillard reaction intermediates and related phytochemicals in black garlic determined by EPR and HPLC analyses. Molecules.

[11] El-Fiki A., Adly M. (2020). Morphological, molecular, and organosulphur compounds characterization in irradiated garlic (Allium sativum) by GC–MS and SCoT markers. J. Radiat. Res. Appl. Sci..

[12] Cui S., Ling P., Zhu H., Keener H.M. (2018). Plant pest detection using an artificial nose system: a review. Sensors.

[13] Da Silva Ferreira M.V., Junior J.L.B., Kamruzzaman M., Barbin D.F. (2023). Low-cost electronic-nose (LC-e-nose) systems for the evaluation of plantation and fruit crops: recent advances and future trends. Anal. Methods.

[14] Makarichian A., Chayjan R.A., Ahmadi E., Zafari D. (2022). Early detection and classification of fungal infection in garlic (A. sativum) using electronic nose. Comput. Electron. Agric..

[15] Rasekh M., Karami H., Kamruzzaman M., Azizi V., Gancarz M. (2023). Impact of different drying approaches on VOCs and chemical composition of Mentha spicata L. essential oil: a combined analysis of GC/MS and E-nose with chemometrics methods. Ind. Crop. Prod..

[16] Seesaard T., Goel N., Kumar M., Wongchoosuk C. (2022). Advances in gas sensors and electronic nose technologies for agricultural cycle applications. Comput. Electron. Agric..

[17] Yuan H., Chen X., Shao Y., Cheng Y., Yang Y., Zhang M., Hua J., Li J., Deng Y., Wang J. (2019). Quality evaluation of green and dark tea grade using electronic nose and multivariate statistical analysis. J. Food Sci..

[18] Fuentes Y.M.O., Ortiz J.C.D., Chávez E.C., Castillo F.D.H., Olivas A.F., Morales G.G., Martínez O.V., Guerra R.R. (2013). The first report of Fusarium proliferatum causing garlic bulb rots in Mexico. Afr. J. Agric. Res..

[19] Li C., Schmidt N.E., Gitaitis R. (2011). Detection of onion postharvest diseases by analyses of headspace volatiles using a gas sensor array and GC-MS. LWT--Food Sci. Technol..

[20] Espitia P.J., Fuenmayor C.A., Otoni C.G. (2019). Nanoemulsions: synthesis, characterization, and application in bio-based active food packaging. Compr. Rev. Food Sci. Food Saf..

[21] Dhalsamant K., Dash S.K., Bal L.M., Sahoo N.R. (2018). Effect of natural antimicrobials (Clove and Garlic) on shelf life and quality of mushroom (Volvariella volvacea) under modified atmosphere. J. Packaging Technol. Res..

[22] Coklar H., Akbulut M., Kilinc S., Yildirim A., Alhassan I. (2018). Effect of freeze, oven and microwave pretreated oven drying on color, browning index, phenolic compounds and antioxidant activity of hawthorn (Crataegus orientalis) fruit. Not. Bot. Horti Agrobot. Cluj-Napoca.

[23] Gholami R., Ahmadi E., Ahmadi S. (2020). Investigating the effect of chitosan, nanopackaging, and modified atmosphere packaging on physical, chemical, and mechanical properties of button mushroom during storage. Food Sci. Nutr..

[24] Lucas P.W., Copes L., Constantino P.J., Vogel E.R., Chalk J., Talebi M., Landis M., Wagner M. (2012). Measuring the toughness of primate foods and its ecological value. Int. J. Primatol..

[25] Mohsenin N.N., Mohsenin N. (1987).

[26] Riudavets J., Pons M.J., Messeguer J., Gabarra R. (2018). Effect of CO2 modified atmosphere packaging on aflatoxin production in maize infested with Sitophilus zeamais. J. Stored Prod. Res..

[27] Booth I.R., Kroll R.G. (1989).

[28] Gsaller F., Furukawa T., Carr P.D., Rash B., Jöchl C., Bertuzzi M., Bignell E.M., Bromley M.J. (2018). Mechanistic basis of pH-dependent 5-flucytosine resistance in Aspergillus fumigatus. Antimicrob. Agents Chemother..

[29] Palmero D., De Cara M., Iglesias C., Moreno M., Gonzalez N., Tello J. (2010). First report of Fusarium proliferatum causing rot of garlic bulbs in Spain. Plant Dis..

[30] Vylkova S., Carman A.J., Danhof H.A., Collette J.R., Zhou H., Lorenz M.C. (2011). The fungal pathogen Candida albicans autoinduces hyphal morphogenesis by raising extracellular pH. mBio.

[31] Sarwar S., Netzel G., Netzel M.E., Mereddy R., Phan A.D.T., Hong H.T., Cozzolino D., Sultanbawa Y. (2021). Impact of curcumin-mediated photosensitization on fungal growth, physicochemical properties and nutritional composition in Australian grown strawberry. Food Anal. Methods.

[32] Siddiq R., Auras R., Siddiq M., Dolan K.D., Harte B. (2020). Effect of modified atmosphere packaging (MAP) and NatureSeal® treatment on the physico-chemical, microbiological, and sensory quality of fresh-cut d'Anjou pears. Food Packag. Shelf Life.

[33] Paulsen E., Barrios S., Baenas N., Moreno D.A., Heinzen H., Lema P. (2018). Effect of temperature on glucosinolate content and shelf life of ready-to-eat broccoli florets packaged in passive modified atmosphere. Postharvest Biol. Technol..

[34] Barikloo H., Ahmadi E. (2018). Effect of nanocomposite-based packaging and chitosan coating on the physical, chemical, and mechanical traits of strawberry during storage. J. Food Meas. Char..

[35] Shen F., Wu Q., Liu P., Jiang X., Fang Y., Cao C. (2018). Detection of Aspergillus spp. contamination levels in peanuts by near infrared spectroscopy and electronic nose. Food Control.

[36] Cui S., Cao L., Acosta N., Zhu H., Ling P.P. (2021). Development of portable e-nose system for fast diagnosis of whitefly infestation in tomato plant in greenhouse. Chemosensors.

